# Perceived Stress Levels in Adult Patients With Uveitis

**DOI:** 10.3389/fpsyt.2019.00916

**Published:** 2020-01-08

**Authors:** Rafael S. Grajewski, Anna C. Boelke, Werner Adler, Arina Pape, Falk Schroedl, Arno Hueber, Christian Albus, Frank Vitinius, Ludwig M. Heindl

**Affiliations:** ^1^Department of Ophthalmology, University of Cologne, Cologne, Germany; ^2^Department of Medical Informatics, Biometry, and Epidemiology, Friedrich-Alexander University Erlangen-Nuernberg, Erlangen, Germany; ^3^Department of Ophthalmology and Institute of Anatomy and Cell Biology, Paracelsus Medical University, Salzburg, Austria; ^4^ Department of Psychosomatic Medicine and Psychotherapy, University of Cologne, Cologne, Germany

**Keywords:** uveitis, stress, Perceived Stress Questionnaire, inflammatory disease, disease activity

## Abstract

**Background:** The aim of this study was to examine perceived stress levels in adult patients with uveitis.

**Patients and Methods:** One hundred seventy-three adult consecutive uveitis patients (age range 18 to 85 years) were analyzed in a cross-sectional design for their perceived stress, according to the Perceived Stress Questionnaire (PSQ). Stress levels were classified into normal stress, moderate stress, and high stress.

**Results:** In the majority of uveitis patients a normal stress level (82%) within the last 2 years was detected. In a subgroup analysis, perceived stress of the patients with active uveitis compared with patients with non-active uveitis was significantly higher within the last 2 years (n=80 active/n = 45 non-active; p = 0.005).

**Conclusions:** Overall 18% of the uveitis patient had raised perceived stress, similar to the general population but patients with active uveitis were significantly more stressed. Therefore, consideration of stress levels may be important in the therapy of uveitis patients.

## Introduction

Vision is the most important sense in humans for obtaining environmental information. In fact, about 40% of our sensory input is visual and the processing of visual information occupies about 50% of our cerebral cortex activity (1). Therefore, actual or threatened vision loss may impose a significant mental strain on many ophthalmological patients.

Uveitis is a major sight-threatening disease that is defined by intraocular inflammation, usually of autoimmune or infectious etiology. Depending on anatomic localization, uveitis is classified into anterior, intermediate, posterior, and panuveitis. The symptoms of uveitis may vary including pain, photophobia, redness, epiphora, and importantly blurred vision. Treatment consists of anti-inflammatory or anti-infectious agents, depending on the underlying etiology. Whereas some forms of uveitis are self-limited, other forms can lead to complete blindness and even death if an underlying systemic disorder remains undetected (2).

In the uveitis service of our department of ophthalmology many uveitis patients report an association between stress and uveitis. They perceive stress preceding the beginning or the recurrence of uveitis.

Two different aspects relationships between stress and uveitis should be distinguished: stress may be a risk factor for inducing the onset or recurrence of an episode of uveitis: and stress may be induced by the onset of uveitis itself.

There are only few studies that have analyzed the association between stress during uveitis, with inconsistent findings (3). Furthermore, these studies are difficult to compare, as they examined different aspects of stress and did not use the terms stress and distress in a consistent manner. On a conceptual level, stress needs to be differentiated from the results of the stress experience, which is often called distress. Stress is a phenomenon of daily life, while distress or anxiety or depressive symptoms refer to psychopathology and psychiatry. In our study, we investigate stress in this former sense by using the Perceived Stress Questionnaire (PSQ). To differentiate these aspects prospective studies are necessary but there are few prospective studies (4, 5). Four studies showed a positive relationship: in a study by Carrim et al. (4), a correlation between stress (from our point of view it is distress instead of stress) and the frequency of recurrences of acute anterior uveitis in susceptible persons has been described using the General Health Questionnaire (GHQ)—an instrument which assessed psychopathology and the subjective experience of stress—and the Social Readjustment Rating Questionnaire (SRRQ). In another study by Franke et al. (5) it was assumed that the beginning of uveitis is distress-induced and that uveitis causes a lower quality of life. Franke et al. (6) used the Brief Symptom Inventory to assess psychological distress. Others, have found that 57.9% of 171 patients with HLA-B27-associated anterior uveitis specified psychological distress as an activator for recurrence and 34.5% named specific life events (6).

The recurrence rate of uveitis after an earthquake in the Hanshin-Awaji (Kobe) area of Japan was significantly higher than before the earthquake (10 *vs.* 3%) (7). The authors suggest that the higher recurrence rate may be caused by stress that is based on the abrupt changes in living conditions after the earthquake. In a recent study by Berlinberg et al., using the Cohen 10-item Perceived Stress Scale (PSS-10), stress was higher in patients with uveitis but with no difference between recently active and controlled uveitis (8).

However, there are also a few studies that report no correlation between stress and uveitis. For example, one study found that the recurrence of idiopathic acute anterior uveitis was not stress-induced in their patient sample (9). The psychological questionnaires that were used in this study were the Holmes and Rahe Stressful Life Events Scale and the Spielberger State-Trait Anxiety Inventory (STAI). There is an overlap between the state and the trait anxiety subscales of STAI and stress but STAI is not a specific stress assessment instrument.

Another study, using the Paykel scale and symptom distress checklist, reported no association between stressful life events or psychological distress and idiopathic recurrent anterior uveitis (10). None of these studies measured perceived stress with the PSQ published by Levenstein et al. (11), and revised and translated into German by Fliege et al. (12, 13). Furthermore, neither study analyzed systematically the potential relationship of stress and uveitic disease activity. The PSQ is a validated and frequently used instrument to measure subjective stress that was originally designed in English and has been translated to Italian, German, Spanish, and Chinese, with validation in populations such as psychiatric inpatients and outpatients, nursing students, health workers, psychosomatic patients, and health adults (11–18). Therefore, the aim of our study was to measure for the first time perceived stress levels in adult uveitis patients using the PSQ, and to examine their association with anatomic location, etiology, age, visual acuity, and disease activity.

## Patients and Methods

Between March 1 and April 30, 2015 all consecutive adult patients (age of 18 years or above) of a databank starting January 1 2012 (n = 527) and including all uveitis patients from the uveitis service at the Department of Ophthalmology, University Hospital of Cologne, Germany, with the following inclusion criteria were mailed by us and asked to participate in our cross-sectional, non-randomized questionnaire-based single-center study:

1. confirmed diagnosis of uni- or bilateral uveitis
2. age at onset of uveitis of 18 years or above.

One hundred seventy-three patients completely filled out the PSQ questionnaires, giving a response rate of 32.8%.

These 173 patients were included in the present retrospective analysis of clinical data performed in conformance with the tenets of the Declaration of Helsinki and approved by our Institutional review board and ethics committee (study number: 14-349).

The patients had not been informed about the study during their original clinical treatment. Informed consent was obtained from all individual participants included in the study and a psychometric postal survey was received by these ophthalmic patients at one measure point.

The three age ranges (18–44, 45–64, > 65 years) were based on clinical practice as different uveitis forms are age dependent, as described before (2).

Uveitis is usually a chronic disease with active and inactive periods. Clinical characteristics could be determined which are not representative of the disease activity. For the whole sample (n = 173) we have data for the clinical characteristics, for a subgroup data regarding the disease activity. Therefore, in a subgroup analysis another criterion was known clinical activity based on retrospective analysis of the charts of the past 2 years defined by the presence of cells in the anterior chamber or vitreous and/or retinal or choroidal inflammation (2, 17). The activity of uveitis (active *versus* inactive) of the last 4 weeks (113 patients) and 2 years (125 patients) before the date of filling the questionnaire could be determined in this subgroup analysis. Data were available from our charts and treating physicians.

At original assessment all patients underwent a comprehensive ophthalmic examination, including measurement of Snellen uncorrected visual acuity and best spectacle-corrected visual acuity (BSCVA). When visual acuity was different between the two eyes, the better visual acuity was considered. External eye examination, slit-lamp biomicroscopy, Goldmann applanation tonometry, and dilated funduscopy were performed, as described previously (2, 15–17).

Standardization of uveitis nomenclature (SUN) criteria were used to classify the uveitis patients according to the primary anatomic site of inflammation: anterior uveitis, intermediate uveitis, posterior uveitis, and panuveitis (19). Patients with intermediate uveitis and significant “spill over” of cells into the anterior chamber or concomitant mild anterior uveitis were classified as intermediate uveitis when the site of maximal inflammation was observed in the vitreous or in the pars plana. Furthermore, the time sequence of the uveitis disease was assessed for all patients. The onset of uveitis was described either as *sudden* or *insidious*. The duration of an attack of uveitis was documented either as *limited*, if it was 3 months or less in duration or as *persistent*, if it was greater than 3 months in duration. The course of uveitis was recorded either as *acute*, if the episode was characterized by sudden onset and limited duration; as *recurrent*, if repeated episodes were separated by periods of inactivity without treatment in at least 3-months duration; or as *chronic*, if there was persistent uveitis with prompt relapse in less than 3 months after discontinuing treatment. Morphologically, uveitis was classified as granulomatous, if large, “mutton-fat” keratic precipitates, Koeppe and/or Busacca iris nodules, and/or optic disk and choroidal granulomas were detected (2, 18).

### Screening for Systemic Disease Associations

All patients were screened for systemic disease associations using detailed clinical history, chest x-ray, blood pressure measurement, urine analysis, complete blood cell count with differential, erythrocyte sedimentation rate, as well as serum analyses for angiotensin-converting enzyme (and lysozyme in patients on angiotensin-converting enzyme inhibitors), sIL-2R, lues, and borrelia, as described previously (2). Using a tailored approach and according to individual clinical suspicion, further investigations were performed, as described previously (2).

The diagnosis of specific manifestations and etiologies of uveitis was based on the clinical picture and on classification according to international criteria [referenced in (2)]. Therefore, patients were grouped into 1) infectious uveitis, 2) specific clinical entity, 3) uveitis associated with systemic disease, and 4) idiopathic uveitis in cases in which a specific diagnosis, either ocular or systemic, could not be made.

### The Perceived Stress Questionnaire

The PSQ-20 is a self-rating instrument which assesses the subjective experience of perceived stress and the existence of external stressors. The questionnaire consisted of 20 questions (see **Appendix** in **Data Sheet 1**) asking how often the particular statements applied. The possible answers included: almost never (yield 1 point in the evaluation), sometimes (2 points), often (3 points), and usually (4 points). Some statements indicated the missing of stress (e.g., “you feel calm”). The PSQ-20 values ranged from 0 (lowest perceived stress) to 1 (highest perceived stress), and included four categories: “worries,” “tension,” and “joy” represented internal stress, and “demands” represented external stressors (13).

Every category consisted of five statements. The first category (*worries*) comprised trouble, anxiety about the future, despair, and frustration. The second category (*tension*) included restlessness, fatigue, and the absence of relaxation. The third category (*joy*) asked for the favorable feelings of challenge, enjoyment, power, and safety. The last category (*demands*) explored external demands such as the lack of time, pressure, and overload. The positively coded category (joy) had to be inversed to build the PSQ score (13). We transformed the individual categories linear to values from 0 to 100.

We used the German translated version of the PSQ-20 (13). The questionnaire PSQ-20 was validated in a German population by Fliege et al. (12). In this version, Cronbach's alpha was not less than 0.85 and reliability not less than 0.8 regarding overall scores. In our sample, Cronbach's alpha for scales worries, tension, joy, and demands (five items each) was 0.84, 0.85, 0.82, and 0.81, respectively for W4 and 0.87, 0.84, 0.84, 0.84 for J2. All values indicate good internal consistency and reliability of the questionnaire.

The patients received per post at the same time both versions of the PSQ-20: The PSQ-20 W4 and the PSQ-20 J2. The PSQ-20 W4 (4 Wochen = 4 weeks) deals with the last 4 weeks and the PSQ-20 J2 (2 Jahre = 2 years) deals with the last 2 years of life. As both tests (PSQ-20 W4 and PSQ-20 J2) are identical beside the time period tested, and because both tests correlated highly in our sample (r = .87), we decided to include only the PSQ-20 J2 in the primary analysis, because it covers a longer time period. Data on the PSQ-20 W4 were included in the Supplemental Data (**Tables 1S–5S**).

### Statistical Analyses

Commercial software (SPSS Statistics version 21.0 for Windows; SPSS Inc., Chicago, IL, USA) used for all statistical analyses. Using mean values of PSQ-20 W4 and PSQ-20 J2 and standard deviation (SD) two cut-off scores to classify the perceived stress into three levels were determined similar to a PSQ study in a Swedish population: low level (≤mean + 1SD), moderate level (> mean + 1 SD and mean < +2 SD), and high level (> mean + 2SD).^19^ The cut-off scores were >0.6378 and < 0.8376 (PSQ-20 W4) respectively >0.6516 and <0.8643 (PSQ-20 J2) for moderately increased stress levels and >0.8376 (PSQ-20 W4) respectively >0.8643 (PSQ-20 J2) for high stress levels.

The association of the PSQ scores with the patient demographics and clinical characteristics was determined using the Mann-Whitney U test and the Kruskal-Wallis test.

Odds ratios showing the association between risk factors and normal or increased (moderate and high) stress level are calculated with the help of generalized estimating equation models. Using these models, measurements of both time points W4 and J2 can be considered but only one odds ratio is calculated for each risk factor.

To explore the association between the visual acuity with the PSQ parameters we used the Pearson correlation. p value of less than 0.05 was considered statistically significant.

## Results

### Patient Demographics and Clinical Characteristics

The anatomic location of the uveitis in our patients was anterior in 49%, intermediate and posterior each in 23%, and panuveitis in 5% (**Table 1**) consistent with the distribution from other studies [summarized in [2]].

**Table 1:** Patient demographics of 173 patients with anterior, intermediate, posterior, and panuveitis.

	Total	Anterior uveitis	Intermediate uveitis	Posterior uveitis	Panuveitis
	(*n = 173*)	*(n = 85)*	*(n = 39)*	*(n = 40)*	*(n = 9)*
**Gender**					
Female, n (%)	111 (64%)	52 (61%)	28 (72%)	26 (65%)	5 (56%)
Male, n (%)	62 (36%)	33 (39%)	11 (28%)	14 (35%)	4 (44%)
**Age at onset (years)**					
Mean ± SD (range)	49 ± 16 (18–85)	50 ± 14 (22–80)	48 ± 17 (19–85)	46 ± 17 (21–84)	54 ± 19 (18–74)
18–24, n (%)	8 (5%)	1 (1%)	2 (5%)	4 (10%)	1 (11%)
25–44, n (%)	64 (37%)	29 (34%)	17 (44%)	16 (40%)	2 (22%)
45–64, n (%)	64 (37%)	40 (47%)	10 (26%)	12 (30%)	2 (22%)
≥65, n (%)	37 (21%)	15 (18%)	10 (26%)	8 (20%)	4 (44%)
**Ethnicity**					
Black, n (%)	3 (2%)	2 (2%)	–	–	1 (11%)
European, n (%)	155 (90%)	76 (89%)	38 (97%)	34 (85%)	7 (78%)
Middle East, n (%)	3 (2%)	2 (2%)	–	–	1 (11%)
Mediterranean ancestry, n (%)	12 (7%)	5 (6%)	1 (3%)	6 (15%)	–
**Ocular involvement**					
Unilateral, n (%)	75 (43%)	43 (51%)	10 (26%)	20 (50%)	2 (22%)
Bilateral, n (%)	98 (57%)	42 (49%)	29 (74%)	20 (50%)	7 (78%)
**Onset of uveitis**					
Sudden, n (%)	116 (67%)	63 (74%)	11 (28%)	34 (85%)	8 (89%)
Insidious, n (%)	57 (33%)	22 (26%)	28 (72%)	6 (15%)	1 (11)
**Duration of an attack of uveitis**					
Limited^†^, n (%)	110 (64%)	62 (73%)	16 (41%)	29 (73%)	3 (33%)
Persistent^‡^, n (%)	63 (36%)	23 (27%)	23 (59%)	11 (28%)	6 (67%)
**Course of uveitis**					
Acute^§^, n (%)	21 (12%)	11 (13%)	2 (5%)	6 (15%)	2 (22%)
Recurrent^$^, n (%)	94 (54%)	51 (60%)	15 (39%)	25 (63%)	3 (33%)
Chronic^#^, n (%)	58 (34%)	23 (27%)	22 (56%)	9 (23%)	4 (44%)
**Type of inflammation**					
Granulomatous, n (%)	14 (8%)	11 (13%)	2 (5%)	–	1 (11%)
Non-granulomatous, n (%)	159 (92%)	74 (87%)	37 (95%)	40 (100%)	8 (89%)
**Etiology of uveitis**					
Infectious uveitis, n (%)	23 (13%)	9 (11%)	–	14 (35%)	–
Specific clinical entity, n (%)	50 (29%)	28 (33%)	–	21 (53%)	1 (11%)
Uveitis associated with systemic disease, n (%)	29 (17%)	12 (14%)	14 (36%)	1 (3%)	2 (22%)
Idiopathic uveitis, n (%)	71 (41%)	36 (42%)	25 (64%)	4 (10%)	6 (67%)
**Visual acuity**					
Mean ± SD (range)	0.85 ± 0.29 (0.04–1.6)	0.86 ± 0.27 (0.05–1.35)	0.81 ± 0.34 (0.1–1.6)	0.89 ± 0.28(0.04–1.6)	0.74 ± 0.34 (0.1–1.25)
<0.1, n (%)	2 (1%)	1 (1%)	–	1 (3%)	–
0.1–0.4, n (%)	15 (9%)	5 (6%)	7 (18%)	2 (5%)	1 (11%)
0.5–1, n (%)	132 (76%)	64 (75%)	27 (69%)	34 (85%)	7 (78%)
>1, n (%)	17 (10%)	10 (12%)	4 (10%)	2 (5%)	1 (11%)

SD, standard deviation.

^†^≤ 3 months duration.

^‡^> 3 months duration.

^§^Episode characterized by sudden onset and limited duration.

^$^Repeated episodes separated by periods of inactivity without treatment ≥3 month in duration.

^#^Persistent uveitis with relapse in <3 month after discontinuing treatment.

The etiology of the uveitis comprised infectious uveitis in 13%, specific clinical entity in 29%, uveitis associated with systemic disease in 17%, and idiopathic uveitis in 41%.

The mean age at onset of uveitis patient was 49.09 ± 15.67 years (range 18 to 85 years). Patients were divided into three age groups: 18 to 44 years including 7,264 patients (42%), 45–64 years including 64 patients (37%), and >65 years including 37 patients (21%).

The mean visual acuity was 0.85 + 0.29 (range 0.04–1.6). Ten percent of patients had a visual acuity of <0.1–0.4, 76% a visual acuity of 0.5–1.0, and 10% a visual acuity of >1.0.

Further demographic and clinical parameters including gender, ethnicity, laterality of ocular involvement, onset, duration of an attack and course of uveitis, and type of inflammation are summarized in **Table 1**.

### Perceived Stress Levels in Adult Uveitis Patients

For the previous 2 years (J2), 82% of the uveitis patients had a normal level of perceived stress (PSQ-20 J2: ≤ 0.6516). Moderately increased levels of perceived stress (PSQ-20 J2: > 0.6516 and < 0.8643) were found in 16% (J2). Highly increased stress levels were found in 2% (J2) (PSQ-20 J2 > 0.8643).

For the previous 4 weeks (W4) 82% of the uveitis patients had a normal level of perceived stress (PSQ-20 W4 ≤ 0.6378). Moderately increased levels of perceived stress (PSQ-20 W4 > 0.6378 and < 0.8376) were found in 18% (W4). Highly increased stress levels were found in 1% (W4) (PSQ-20 W4 > 0.8376).

### Association of Perceived Stress Levels With Clinical Characteristics of Uveitis

The anatomic location of uveitis (**Table 2**) was not significantly associated with *worries* (p = 0.667), *tension* (p = 0.880), *joy* (p = 0.667), or *demands* (p = 0.050). Furthermore, there was no significant association of the anatomic location of uveitis with the overall scores of perceived stress (p = 0.549).

**Table 2:** Perceived Stress Questionnaire values of 173 uveitis patients in dependence of the anatomic location of inflammation.

	Anterior uveitis	Intermediate uveitis	Posterior uveitis	Panuveitis	p-value
	*(n = 85)*	*(n = 39)*	*(n = 40)*	*(n = 9)*	
**Worries J2**					
Mean ± SD	37 ± 26	40 ± 24	34 ± 27	36 ± 26	0.667
**Tension J2**					
Mean ± SD	51 ± 23	51 ± 25	49 ± 26	42 ± 34	0.880
**Joy J2**					
Mean ± SD	51 ± 26	55 ± 25	57 ± 24	59 ± 24	0.667
**Demands J2**					
Mean ± SD	47 ± 23	38 ± 29	39 ± 23	33 ± 24	0.050
**PSQ-20 J2**					
Mean ± SD	0.46 ± 0.21	0.43 ± 0.22	0.41 ± 0.21	0.38 ± 0.22	0.549

SD, standard deviation.

The etiology of uveitis (**Table 3**) was not significantly associated with *worries* (p = 0.776), *tension* (p = 0.346), *joy* (W4: p = 0.254), or demands (p = 0.345). Furthermore, there was no significant association of the etiology of uveitis with the overall scores of perceived stress (p = 0.507).

**Table 3:** Perceived Stress Questionnaire values of 173 uveitis patients in dependence of the etiology of uveitis.

	Infectious uveitis	Specific clinical entity	Uveitis associated with systemic disease	Idiopathic uveitis	p-value
	*(n = 23)*	*(n = 50)*	*(n = 29)*	*(n = 71)*	
**Worries J2**					
Mean ± SD	39 ± 29	34 ± 26	37 ± 23	38 ± 26	0.776
**Tension J2**					
Mean ± SD	58 ± 23	47 ± 23	48 ± 20	50 ± 28	0.346
**Joy J2**					
Mean ± SD	46 ± 21	55 ± 25	60 ± 21	53 ± 28	0.254
**Demands J2**					
Mean ± SD	45 ± 19	45 ± 23	35 ± 27	43 ± 26	0.345
**PSQ-20 J2**					
Mean ± SD	0.49 ± 0.18	0.43 ± 0.2	0.4 ± 0.19	0.44 ± 0.24	0.507

SD, standard deviation.

The age of uveitis patients (**Table 4**) was significantly positively associated with worries (p = 0.002, respectively) and joy (p = 0.001), but negatively associated with tension (p = 0.003), and demands (p < 0.001). Furthermore, there was a significant negative association of the age of uveitis patients with the overall scores of perceived stress (p < 0.001).

**Table 4:** Perceived Stress Questionnaire values of 173 uveitis patients in dependence of the age groups.

	18–44 years	45–64 years	≥ 65 years	p-value
	*(n = 72)*	*(n = 64)*	*(n = 37)*	
**Worries J2**				
Mean ± SD	39 ± 26	41 ± 26	24 ± 21	0.002^a^
**Tension J2**				
Mean ± SD	54 ± 24	53 ± 23	36 ± 25	0.003^a^
**Joy J2**				
Mean ± SD	51 ± 24	49 ± 26	67 ± 24	0.001^a^
**Demands J2**				
Mean ± SD	51 ± 22	46 ± 22	20 ± 20	<0.001^a^
**PSQ-20 J2**				
Mean ± SD	0.48 ± 0.2	0.48 ± 0.2	0.28 ± 0.18	<0.001^a^

SD, standard deviation.

a Significant after Benjamini-Hochberg correction for multiple testing.

The global scores of the PSQ-20 (J2) were highest in the 18–44 and the 45–64 years age groups. The oldest patients (>65 years) perceived the lowest stress.

The categories *worries, tension*, and *demands* in the PSQ-20 J2 were lowest in the oldest patient group (>65 years). Demands were highest in the 18–44 years old patient group. Joy was highest in the oldest patient group (>65 years).

The visual acuity of uveitis patients (**Table 5**) was not significantly associated with worries (p = 0.922), tension (p = 0.154), and joy (p = 0.808). In contrast, visual acuity was significantly associated with demands (p < 0.001). There was no significant association of visual acuity of uveitis patients with the overall scores of perceived stress levels (p = 0.095).

**Table 5:** Perceived Stress Questionnaire values of 173 uveitis patients in dependence of the visual acuity.

	<0.1–0.4	0.5–1.0	>1.0	p-value
	(*n = 24*)	(*n = 132)*	(*n = 17*)	
**Worries J2**				
Mean ± SD	31 ± 27	37 ± 25	41 ± 26	0.425
**Tension J2**				
Mean ± SD	35 ± 22	52 ± 24	47 ± 28	0.019
**Joy J2**				
Mean ± SD	61 ± 23	53 ± 26	55 ± 26	0.533
**Demands J2**				
Mean ± SD	20 ± 17	45 ± 24	48 ± 24	<0.001^a^
**PSQ-20 J2**				
Mean ± SD	0.31 ± 0.18	0.45 ± 0.21	0.45 ± 0.23	0.033

SD, standard deviation.

a Significant after Benjamini-Hochberg correction for multiple testing.

The category demand was highest in the >1.0 vision groups and lowest in the <0.1–0.4 vision group.

**Table 6S** shows odds ratios for type of uveitis, etiology, age, visual acuity and disease activity when comparing patients with normal to those with increased (moderate or high) stress level. Only age group shows a significant effect: the risk of increased stress level is significantly reduced in patients older than 65 years when compared to younger patients (OR=0.102; 95%CI: 0.015 – 0.706; P=0.021).

### Subgroup Analysis

In a subgroup analysis another criterion was known clinical activity based on retrospective analysis of the charts of the past 2 years defined by the presence of cells in the anterior chamber or vitreous and/or retinal or choroidal inflammation. One hundred twenty-five patients fulfilled this criterion regarding the previous 2 years (PSQ-20 J2).

The status of clinical activity (active *versus* inactive) of uveitis (**Table 6**) within the last 2 years was significantly associated with worries (related to the last 2 years: J2: p = 0.001. Furthermore, there was a significant association of clinical activity of uveitis in the last 2 years with the overall scores of perceived stress (p = 0.005).

**Table 6:** Perceived Stress Questionnaire values of 125 (uveitis activity J2) uveitis patients in dependence of the disease activity (active *vs*. inactive; last 2 years).

	Active J2	Inactive J2	p-value
	(*n = 80*)	(*n = 45*)	
**Worries J2**			
Mean ± SD	42 ± 26	26 ± 22	0.001^a^
**Tension J2**			
Mean ± SD	55 ± 25	43 ± 25	0.035
**Joy J2**			
Mean ± SD	49 ± 26	59 ± 24	0.042
**Demands J2**			
Mean ± SD	45 ± 25	35 ± 23	0.040
**PSQ-20 J2**			
Mean ± SD	0.48 ± 0.22	0.36 ± 0.20	0.005^a^

SD, standard deviation.

a Significant after Benjamini-Hochberg correction for multiple testing.

### Mixed Regression Models

Detailed results of all five models are given in **Tables 7**–**11**. Overall, in the mixed regression models age has shown to be the most influential variable associated with stress, which is consistent with the finding in the bivariate analysis (p=0.023 (worries), p=0.031 (tension), p=0.003 (joy), p<0.001 (demands), p<0.001 (PSQ20)). Disease activity had significant influence on worries (p=0.044), tension (p=0.006), and PSQ20 (p=0.031), which was also found in the bivariate analysis. However, only the multivariate analysis showed that tension and PSQ20 were significantly reduced when etiology of uveitis was specific clinical entity (tension: beta=-17.03, p=0.012; PSQ20: beta=-12.84, p=0.023) or associated with systemic disease (tension: beta=-18.67, p=0.026; PSQ20: beta=-17.66, p=0.011), in comparison to infectious uveitis. Similarly, joy was significantly increased when etiology was different to infectious uveitis (specific clinical entity: beta=16.77, p=0.012; associated systemic disease: beta=28.73, p=0.001; idiopathic: beta=14.53; p=0.046). Another finding different to the bivariate analysis was the dependency of demands and PSQ20 from the type of uveitis. Both were significantly reduced, when the uveitis type was posterior, instead of anterior (demands: beta=-13.94, p=0.009; PSQ20: beta=-10.51, p=0.031). Additionally, demands was significantly reduced for the type panuveitis instead of anterior uveitis (beta=-19.71, p=0.029). No significant influence of the time point (W4 vs J2) was found in any of the models.

**Table 7:** Regression models of the Perceived Stress Questionnaire parameter demands in dependence of clinical parameters.

	Estimate	95% CI	p-value
**Intercept**	86,79	58.889 – 114.69	<0.001
**Type: intermediate (ref. anterior)**	−9,833	−20.346 – 0.679	0.069
**Type: posterior**	−13,935	−24.225 – −3.645	0.009
**Type: panuveitis**	−19,706	−37.152 – −2.26	0.029
**Etiology: spec. clin. Ent. (ref. infection)**	−7,855	−19.674 – 3.964	0.195
**Etiology: assoc. systemic disease**	−10,169	−24.734 – 4.397	0.174
**Etiology: idiopathic**	−5,039	−17.985 – 7.906	0.447
**Age**	−0,715	−0.991 – −0.44	<0.001
**Visual acuity**	5,5	−8.392 – 19.392	0.439
**Activity: active (ref. inactive)**	1,883	−2.854 – 6.621	0.437
**Time: J2 (ref.: W4)**	−1,666	−4.771 – 1.44	0.295

CI, confidence interval.

**Table 8:** Regression models of the Perceived Stress Questionnaire parameter joy in dependence of clinical parameters.

	Estimate	95% CI	p-value
**Intercept**	1,113	−29.318 – 31.544	0,943
**Type: intermediate (ref. anterior)**	−4,469	−15.936 – 6.997	0,447
**Type: posterior**	10,743	−0.479 – 21.965	0,063
**Type: panuveitis**	7,287	−11.743 – 26.316	0,455
**Etiology: spec. clin. Ent. (ref. infection)**	16,776	3.881 – 29.671	0,012
**Etiology: assoc. systemic disease**	28,732	12.841 – 44.623	0,001
**Etiology: idiopathic**	14,525	0.4 – 28.651	0,046
**Age**	0,463	0.162 – 0.763	0,003
**Visual acuity**	13,248	−1.91 – 28.406	0,09
**Activity: active (ref. inactive)**	−4,347	−9.372 – 0.677	0,092
**Time: J2 (ref.: W4)**	2,047	−1.228 – 5.323	0,223

CI, confidence interval.

**Table 9:** Regression models of the Perceived Stress Questionnaire parameter PSQ20 in dependence of clinical parameters.

	Estimate	95% CI	p-value
**Intercept**	84,01	58.355 – 109.664	<0.001
**Type: intermediate (ref. anterior)**	0,837	−8.831 – 10.505	0.866
**Type: posterior**	−10,512	−19.971 – −1.053	0.031
**Type: panuveitis**	−10,421	−26.465 – 5.624	0.206
**Etiology: spec. clin. Ent. (ref. infection)**	−12,838	−23.715 – −1.961	0.023
**Etiology: assoc. systemic disease**	−17,657	−31.06 – −4.254	0.011
**Etiology: idiopathic**	−9,334	−21.25 – 2.582	0.128
**Age**	−0,473	−0.727 – −0.22	<0.001
**Visual acuity**	−3,989	−16.778 – 8.799	0.542
**Activity: active (ref. inactive)**	4,447	0.441 – 8.454	0.031
**Time: J2 (ref.: W4)**	−1,925	−4.512 – 0.662	0.148

CI, confidence interval.

**Table 10:** Regression models of the Perceived Stress Questionnaire parameter tension in dependence of clinical parameters.

	Estimate	95% CI	p-value
**Intercept**	79,12	48.142 – 110.099	<0.001
**Type: intermediate (ref. anterior)**	3,677	−7.994 – 15.347	0.538
**Type: posterior**	−6,661	−18.088 – 4.767	0.256
**Type: panuveitis**	−4,094	−23.462 – 15.274	0.679
**Etiology: spec. clin. Ent. (ref. infection)**	−17,029	−30.142 – −3.917	0.012
**Etiology: assoc. systemic disease**	−18,671	−34.833 – −2.51	0.026
**Etiology: idiopathic**	−10,913	−25.274 – 3.448	0.139
**Age**	−0,342	−0.648 – −0.036	0.031
**Visual acuity**	0,396	−15.013 – 15.806	0.96
**Activity: active (ref. inactive)**	8,011	2.423 – 13.599	0.006
**Time: J2 (ref.: W4)**	−3,19	−6.902 – 0.522	0.095

CI, confidence interval.

**Table 11:** Regression models of the Perceived Stress Questionnaire parameter worries in dependence of clinical parameters.

	Estimate	95% CI	p-value
**Intercept**	70,153	38.997 – 101.309	<0.001
**Type: intermediate (ref. anterior)**	5,002	−6.739 – 16.743	0.406
**Type: posterior**	−10,187	−21.675 – 1.301	0.085
**Type: panuveitis**	−10,813	−30.298 – 8.672	0.279
**Etiology: spec. clin. Ent. (ref. infection)**	−9,889	−23.098 – 3.319	0.145
**Etiology: assoc. systemic disease**	−13,256	−29.532 – 3.02	0.113
**Etiology: idiopathic**	−7,025	−21.495 – 7.444	0.343
**Age**	−0,362	−0.67 – −0.054	0.023
**Visual acuity**	−8,567	−24.096 – 6.962	0.282
**Activity: active (ref. inactive)**	5,099	0.168 – 10.03	0.044
**Time: J2 (ref.: W4)**	−1,294	−4.484 – 1.897	0.428

CI, confidence interval.

## Discussion

This is the first study to analyze perceived stress levels in uveitis, a major sight-threatening disease, using the PSQ.

We found that perceived stress was significantly higher in patients with active uveitis within the last 2 years in comparison to patients with quiescent uveitis for at least 2 years, respectively. Overall 18% of the uveitis patient had raised perceived stress, similar to the general population but patients with active uveitis were significantly more stressed. Furthermore, the external perceived stress parameter “demands” was significantly negatively associated with visual acuity (defined as best-corrected sharpness of vision). The risk for a moderate or higher stress level however was not increased significantly, when the disease was active (OR = 1.932 but 95% CI = 0.888–4.204 and P = 0.097).

When comparing the classification of the stress levels the values of PSQ-20 W4 and PSQ-20 J2 were similar, indicating that the stress level rarely changed when comparing the last 2 years *versus* the last month.

This result could depend on a recall bias of the patients who could not differentiate exactly between former and acute stress.

A German study explored the perceived stress of the adult German general population (N = 2552) and the results show a prevalence rate for a moderately raised stress level (cut-off value: > 0.45) from 14.5 and 3.1% for a high stress level (cut-off value: 0.6) (14, 19). Thus, 17.6% have an increased stress level. These results are similar to our results, when analyzing all consecutive uveitis patients regardless of clinical activity. When considering the activity of clinical disease there was a significantly higher stress level in patients with active inflammation within the last 2 years (**Table 6**). That suggests that in patients with quiescent disease the moderate stress level of uveitis patients is similar (16–18 *vs.* 14.5%) to the German general population, but patients with active disease have a higher stress level. Lower overall stress levels compared to our results and the results of the German study (14, 19), were found in a study by Bergdahl et al. who used the PSQ to examine the stress level of 1275 Swedish patients from the dental health service registers with the age ranged from 20 to 69 years, that represented the Swedish population. In this study a moderate stress level occurs in 10% and high stress level in 4% of the population (12). These differences may be due to different living conditions in Sweden compared to Germany.

Furthermore, we determined a significant association between perceived stress and the age of uveitis patients. Perceived stress was higher in the age groups with patients between 18 and 44 and 45–64 years. Lowest perceived stress occurred in the oldest age group ≥65 years. The odds ratio shows that the risk for increased stress level is reduced to about 10% in patients older than 65 years (OR = 0.102). Similar results were reported in a German study on a normal population: perceived stress was highest in the age group from 31 to 60 years old, followed by patients from 18 to 30 years and lowest perceived stress was found in patients over 61 years (19). The study of Fliege et al. showed that perceived stress declines in patients over 60 years (13).

In our study we found significant associations between visual acuity and the category “demands” for both the last 4 weeks and the last 2 years. The patients with the best visual acuity (0.5–1.0 and 1.0) perceived highest demands, and patients with lowest visual acuity (<0.1–0.4) the lowest demands. As demands represents the subjective perceived external stressors (e.g., absence of time, pressure, and congestion), this could represent a decrease of demands for patients with visual disability who sometimes may receive more social support. However, the risk for increased stress level was not significantly increased for patients with higher visual acuity.

Murphy et al. have shown that most intermediate uveitis patients can keep their good visual acuity and quality of life (20). This study showed a correlation between the vision in the worse eye and the impairment of the vision related quality of life and an association between the impairment of the general health related quality of life in patients with intermediate uveitis. However, we found no significant association between stress and the anatomic location and etiology of uveitis. Other studies involve only patients with anterior uveitis (4, 5, 7, 10) or they did not provide the anatomic location of the uveitis (6, 8). So we cannot compare our results with these other studies in relation to the anatomic location. Moreover, some other studies did not specify the etiology or they did not show comparisons to other etiologies in relation to the perceived stress. Yamamoto et al. gave information on etiology, but provide no statistical results in relation to the anatomic location and the association with stress (7). Berlinberg et al. (8) compared stress levels in patients with active *versus* inactive uveitis, defined by inflammatory activity within the past 90 days. In contrast to our results that included episodes of activity of the last 2 years, they found no difference between active *versus* controlled uveitis. This highlights the need for further prospective studies because one single factor such as disease activity could be further subdivided dependent on the numbers and activity grades of uveitic recurrences and the duration of disease and observation.

The sample of the current study is comparable with the whole cohort of our uveitis service (2): e.g., subtype of uveitis [53% for anterior, 19% for intermediate, 21% for posterior, and 7% for panuveitis (2) *versus* 49% for anterior, 23% for intermediate, 23% for posterior, and 5% for panuveitis in the current sample of responders], age at onset, laterality, etiology. In the current sample there are 64% female patients in the responder group of the current sample, in the former publication there were 55% female patients.

Stress appears to play an important role for numerous diseases and living conditions including inflammatory bowel disease (21), and multiple sclerosis (22), cardiovascular disease (21) psychosomatic disorders, females after spontaneous abortion, students, and tinnitus patients (13), compared to healthy adults. In our sample patients with active disease had higher stress levels compared with non-active disease.

An interesting finding revealed by multivariate regression analysis was reduced tension and overall PSQ20 stress level, when etiology was specific clinical entity or associated with systemic disease as opposed to an infectious etiology. At this moment, we can only speculate about the significance of this difference. The most common infectious etiologies in our sample were infections with *Toxoplasma gondii* and herpetic viruses (herpes simplex, varicella zoster, and cytomegalovirus). Interestingly, in a recent large-scale study by Burgdorf et al. (23) infections with *Toxoplasma gondii* and cytomegalovirus showed association with serious psychiatric disorders.

Other findings in the bivariate analysis such as the role of age as the most influential variable on stress in our sample, as well as the significant influence on worries, tension, and PSQ20, were confirmed by the mixed regression models.

A limitation of our study is indeed the cross-sectional design implying inclusion of different clinical stages of uveitis such as recent onset *versus* chronic disease. On the other the hand the mixed sample is a strength of the study which allows us to determine differences between the groups.

There are at least two possible causal interactions between stress and uveitis: stress may be a risk factor for inducing the onset of uveitis; or a reaction to the symptoms and limitations imposed by uveitis itself, such as decreased visual acuity. The cross-sectional design of this study means it cannot assess the nature of any cause-effect relationships in the correlations it identifies.

The follow-up assessment had a response rate of nearly a third of the whole clinical sample, of ophthalmic patients who were not informed about the study during their original clinical treatment. We do not know the activity level of uveitis of the non-enrolled patients, which could introduce bias. There are some other potential sources of bias: i.) more stressed patients could be either more or less likely to complete the questionnaire. ii.) More stressed patients could be more likely to present with symptoms and thus more likely to be diagnosed as active. iii.) Uveitis treatment could contribute to perceived stress. For example, systemic treatment with corticosteroids could lead to depressive symptoms or altered mental states.

Treatments as a possible cause of perceived stress could not be adequately assessed due to the retrospective design of our study and the heterogeneous sample of our subgroups (different time points of treatment, different length of treatment, different dosages, different grades of uveitis, different specific entities).

Future research in the uveitis field should use a prospective design to assess possible etiological relationships between disease onset *vs.* activity and acute *vs.* chronic stress or if other factors such as infections may contribute independently and causatively to stress, as discussed above. For example, in the study of Levenstein et al. (24) short-term stress (4 Weeks) assessed with PSQ does not trigger exacerbation in ulcerative colitis, but long-term perceived stress (2 Years) increases the risk of exacerbation over a period of months to years.

In summary, the results tentatively suggest that there is an association of perceived stress and uveitis depending on clinical activity. Patient with quiescent periods of at least 2 years did not differ from the general population regarding their stress levels. Therefore, future prospective studies should determine whether the patients with active and recurrent uveitis may benefit from therapeutic psychosomatic/behavioral interventions in addition to their anti-inflammatory therapy.

## Ethics Statement

All procedures performed in studies involving human participants were in accordance with the ethical standards of the institutional and/or national research committee and with the 1964 Helsinki declaration and its later amendments or comparable ethical standards. Informed consent was obtained from all individual participants included in the study.

## Author Contributions

RG, CA, FV and LH contributed conception and design of the study. AB and AP organized the database. LH and WA performed the statistical analysis. RG wrote the first draft of the manuscript. FV, AH, FS, LH, and WA wrote sections of the manuscript. All authors contributed to manuscript revision, read and approved the submitted version.

## Funding

No funding was received for this research. Other funding (independently of this study): German Research Foundation (FOR 2240 “(Lymph)Angiogenesis And Cellular Immunity In Inflammatory Diseases Of The Eye”) to RSG and LMH; GR 2647/5-1 to RSG; HE 6743/2-1 and 3-1 to LMH) and GEROK program by the University of Cologne (to RSG and LMH).

## Conflict of Interest

RSG received a speaker honorarium from the companies AbbVie, Allergan and Chugai.

The remaining authors declare that the research was conducted in the absence of any commercial or financial relationships that could be construed as a potential conflict of interest.
