# Supplementary material for: Perceived Stress Levels in Adult Patients With Uveitis
Source: Front Psychiatry. 2020 Jan 8;10:916. doi: 10.3389/fpsyt.2019.00916 (PMC6960170; doi:10.3389/fpsyt.2019.00916)
Supplement: Supplementary file 1 [file DataSheet_1.docx]

**Supplemental Data:**

**Table 1S: PSQ values of 173 uveitis patients in dependence of the anatomic location of inflammation**

|  | **Anterior Uveitis**  *(n=85)* | **Intermediate Uveitis**  *(n=39)* | **Posterior Uveitis**  *(n=40)* | **Panuveitis**  *(n=9)* | **p-value** |
| --- | --- | --- | --- | --- | --- |
| **Worries W4**  Mean ± SD | 36 ± 24 | 39 ± 23 | 34 ± 24 | 38 ± 22 | 0.715 |
| **Tension W4**  Mean ± SD | 49 ± 24 | 51 ± 29 | 49 ± 22 | 48 ± 22 | 0.974 |
| **Joy W4**  Mean ± SD | 53 ± 25 | 52 ± 24 | 54 ± 24 | 52 ± 14 | 0.999 |
| **Demands W4**  Mean ± SD | 46 ± 24 | 39 ± 26 | 41 ± 21 | 33 ± 23 | 0.164 |
| **PSQ-20 W4**  Mean ± SD | 0.44 ± 0.20 | 0.44 ± 0.21 | 0.42 ± 0.19 | 0.42 ± 0.17 | 0.948 |

SD standard deviation.

**Table 2S: PSQ values of 173 uveitis patients in dependence of the etiology of uveitis**

|  | **Infectious uveitis** | **Specific clinical entity** | **Uveitis associated with systemic disease** | **Idiopathic uveitis** | **p-value** |
| --- | --- | --- | --- | --- | --- |
|  | (*n=23*) | (*n=50*) | (*n=29*) | (*n=71*) |  |
| **Worries W4**  Mean ± SD | 40 ± 24 | 32 ± 24 | 37 ± 19 | 38 ± 25 | 0.436 |
| **Tension W4**  Mean ± SD | 54 ± 24 | 47 ± 23 | 49 ± 24 | 50 ± 26 | 0.533 |
| **Joy W4**  Mean ± SD | 44 ± 21 | 57 ± 23 | 57 ± 20 | 51 ± 26 | 0.140 |
| **Demands W4**  Mean ± SD | 42 ± 21 | 47 ± 24 | 36 ± 23 | 42 ± 25 | 0.212 |
| **PSQ-20 W4**  Mean ± SD | 0.48 ± 0.17 | 0.42 ± 0.2 | 0.41 ± 0.17 | 0.45 ± 0.22 | 0.554 |

SD standard deviation.

**Table 3S: PSQ values of 173 uveitis patients in dependence of the age groups**

|  | **18-44 yrs** | **45-64 yrs** | **≥ 65 yrs** | **p-value** |
| --- | --- | --- | --- | --- |
|  | (*n=72*) | (*n=64)* | (*n=37*) |  |
| **Worries W4**  Mean ± SD | 39 ± 24 | 39 ± 24 | 26 ± 20 | 0.009^a^ |
| **Tension W4**  Mean ± SD | 53 ± 23 | 53 ± 24 | 36 ± 24 | 0.001^a^ |
| **Joy W4**  Mean ± SD | 49 ± 22 | 50 ± 25 | 65 ± 23 | 0.002^a^ |
| **Demands W4**  Mean ± SD | 50 ± 22 | 46 ± 21 | 21 ± 20 | <0.001^a^ |
| **PSQ-20 W4**  Mean ± SD | 0.48 ± 0.18 | 0.47 ± 0.2 | 0.3 ± 0.18 | <0.001^a^ |

SD standard deviation

a Significant after Benjamini-Hochberg correction for multiple testing

**Table 4S: PSQ values of 173 uveitis patients in dependence of the visual acuity**

|  | **<0.1-0.4** | **0.5-1.0** | **>1.0** | **p-value** |
| --- | --- | --- | --- | --- |
|  | (*n=24*) | (*n=132)* | (*n=17*) |  |
| **Worries W4**  Mean ± SD | 32 ± 24 | 37 ± 24 | 37 ± 19 | 0.665 |
| **Tension W4**  Mean ± SD | 40 ± 28 | 51 ± 24 | 47 ± 23 | 0.127 |
| **Joy W4**  Mean ± SD | 56 ± 25 | 53 ± 24 | 54 ± 22 | 0.691 |
| **Demands W4**  Mean ± SD | 21 ± 17 | 45 ± 24 | 44 ± 20 | <0.001^a^ |
| **PSQ-20 W4**  Mean ± SD | 0.34 ± 0.2 | 0.45 ± 0.2 | 0.44 ± 0.17 | 0.088 |

SD standard deviation

a Significant after Benjamini-Hochberg correction for multiple testing

**Table 5S (A-C): PSQ values of 113 (uveitis activity W4) uveitis patients in dependence of the disease activity (active vs. inactive; last 4 weeks vs. last 2 years)**

| A | **Active W4** | **Inactive W4** | **p-value** |
| --- | --- | --- | --- |
|  | (*n=29*) | (*n=84*) |  |
| **Worries W4**  Mean ± SD | 43 ± 24 | 32 ± 21 | 0.023 |
| **Tension W4**  Mean ± SD | 62 ± 20 | 45 ± 25 | 0.003^a^ |
| **Joy W4**  Mean ± SD | 45 ± 16 | 56 ± 25 | 0.028 |
| **Demands W4**  Mean ± SD | 49 ± 24 | 39 ± 23 | 0.076 |
| **PSQ-20 W4**  Mean ± SD | 0.52 ± 0.17 | 0.40 ± 0.19 | 0.003^a^ |

SD standard deviation

a Significant after Benjamini-Hochberg correction for multiple testing

| B | **Active J2** | **Inactive J2** | **p-value** |
| --- | --- | --- | --- |
|  | (*n=80*) | (*n=45*) |  |
|  |  |  |  |
| **Worries W4**  Mean ± SD | 41 ± 24 | 28 ± 21 | 0.002^a^ |
| **Tension W4**  Mean ± SD | 53 ± 22 | 45 ± 28 | 0.165 |
| **Joy W4**  Mean ± SD | 49 ± 22 | 57 ± 26 | 0.051 |
| **Demands W4**  Mean ± SD | 46 ± 23 | 36 ± 24 | 0.026 |
| **PSQ-20 W4**  Mean ± SD | 0.48 ± 0.19 | 0.38 ± 0.21 | 0.011^a^ |

SD standard deviation

a Significant after Benjamini-Hochberg correction for multiple testing

| C | **Active W4** | **Inactive W4** | **p-value** |
| --- | --- | --- | --- |
|  | (*n=29*) | (*n=84*) |  |
| **Worries J2**  Mean ± SD | 43 ± 26 | 32 ± 25 | 0.030 |
| **Tension J2**  Mean ± SD | 61 ± 22 | 45 ± 24 | 0.009^a^ |
| **Joy J2**  Mean ± SD | 46 ± 22 | 57 ± 25 | 0.072 |
| **Demands J2**  Mean ± SD | 46 ± 24 | 38 ± 24 | 0.106 |
| **PSQ-20 J2**  Mean ± SD | 0.51 ± 0.19 | 0.40 ± 0.21 | 0.013^a^ |

SD standard deviation

a Significant after Benjamini-Hochberg correction for multiple testing

**Table 6S (A-E): Odds ratios and confidence intervals for increased stress levels in uveitis patients in dependence of age (A), disease activity (B), better visual acuity (C), etiology (D) and type of uveitis (E)**

| A | **OR** | **95% CI** | **p-value** |
| --- | --- | --- | --- |
| **Reference: Age <65 years** | 1 | 32 ± 21 | 0.023 |
| **Age > 65 years** | 0.102 | 0.015-0.706 | 0.021 |

OR odds ratio

CI confidence interval

| B | **OR** | **95% CI** | **p-value** |
| --- | --- | --- | --- |
| **Reference: Inactive** | 1 |  |  |
| **Active** | 1.932 | 0.888-4.204 | 0.097 |

OR odds ratio

CI confidence interval

| C | **OR** | **95% CI** | **p-value** |
| --- | --- | --- | --- |
| **Reference: VA < 0.5** | 1 |  |  |
| **VA 0.5-1.0** | 3.556 | 0.523-24.19 | n.s. |
| **VA >1.0** | 4,148 | 0.466-36.945 | n.s. |

OR odds ratio

CI confidence interval

VA visual acuity

n.s. not significant

| D | **OR** | **95% CI** | **p-value** |
| --- | --- | --- | --- |
| **Reference: Idiopathic uveitis** | 1 |  |  |
| **Infectious uveitis** | 0.985 | 0.419-2.317 | n.s. |
| **Specific clinical entity** | 1.32 | 0.707-2.466 | n.s. |
| **Uveitis associated with systemic disease** | 0.442 | 0.149-1.307 | n.s. |

OR odds ratio

CI confidence interval

n.s. not significant

| E | **OR** | **95% CI** | **p-value** |
| --- | --- | --- | --- |
| **Reference: Anterior uveitis** | 1 |  |  |
| **Intermediate uveitis** | 1.157 | 0.49-2.73 | n.s. |
| **Posterior uveitis** | 0.593 | 0.216-1.627 | n.s. |
| **Panuveitis** | 0 | 0.000-Inf | n.s. |

OR odds ratio

CI confidence interval

n.s. not significant

Inf infinity

APPENDIX

PSQ 20 items:

Scale “worries”

- You are afraid for the future

- You have many worries

- Your problems seem to be piling up

- You fear you may not manage to attain your goals

- You feel frustrated

Scale “tension”

- You feel tense

- You feel rested

- You feel mentally exhausted

- You have trouble relaxing

- You feel calm

Scale “joy”

- You feel you’re doing things you really like

- You enjoy yourself

- You are lighthearted

- You are full of energy

- You feel safe and protected

Scale “demands”

- You have too many things to do

- You have enough time for yourself

- You feel under pressure from deadlines

- You feel you’re in a hurry

- You feel that too many demands are being made on you
